# Pneumothorax as a Prelude: Unveiling Concurrent HIV and Pneumocystis jirovecii Pneumonia in a Diagnostic Journey

**DOI:** 10.7759/cureus.60697

**Published:** 2024-05-20

**Authors:** Audrey Bui, Austin Vaughn, Douglass Sherard

**Affiliations:** 1 Medicine, Lake Erie College of Osteopathic Medicine, Bradenton, USA; 2 Radiology, Lake Erie College of Osteopathic Medicine, Bradenton, USA; 3 Interventional Radiology, Ascension St. Vincent's Hospital, Bradenton, USA

**Keywords:** human immunodeficiency virus (hiv), opportunistic pathogen, secondary pneumothorax, hiv aids, pneumocystis pneumonia (pcp)

## Abstract

*Pneumocystis jirovecii* pneumonia (PCP) is a life-threatening condition found in immunocompromised individuals, especially in human immunodeficiency virus (HIV) positive patients. Here, we report a case of PCP in a presumably immunocompetent 25-year-old male patient who presented with a one-month history of chest pain, dyspnea, and a nonproductive cough with recent development of night sweats. The patient recently immigrated to the United States without any known medical or family history. A chest radiograph revealed moderate pneumothorax for which a chest tube was placed. A chest computed tomography (CT) scan revealed diffuse lung disease with multiple thin- and thick-walled cystic lesions on a background of diffuse ground-glass opacities. Based on these radiologic findings and subsequent positive HIV serology, there was a high suspicion of PCP. Bronchoalveolar lavage was performed, and PCR for *Pneumocystis jirovecii* was positive. Appropriate treatment was initiated, and the patient recovered well. Through this report, we aim to highlight the importance of recognizing the various clinical and radiologic findings of PCP even in patients with no overt risk factors. Prompt and targeted treatment could mitigate morbidity and mortality associated with this opportunistic pathogen.

## Introduction

*Pneumocystis jirovecii* pneumonia (PCP) represents a model opportunistic infection, demonstrating the complex interplay between pathogen and host immune system, particularly in immunocompromised individuals. Traditionally linked with patients suffering from acquired immunodeficiency syndrome (AIDS), PCP has seen an epidemiological shift and is now also frequently diagnosed in patients undergoing immunosuppressive therapy for malignancies, those with rheumatologic disorders, and those receiving post-transplantation care [1]. While advances in prophylactic measures and antiretroviral therapies have led to a decline in incidence among patients living with HIV, the broader application of potent immunosuppressive regimens has unveiled a diverse spectrum of PCP presentations, challenging clinicians and radiologists alike [2].

This case report aims to underscore the multifaceted clinical and radiologic manifestations of PCP, which range from subtle and nonspecific symptoms to acute respiratory distress, reflecting the pathogen's capability to exploit varied states of immune compromise. Clinically, PCP can masquerade as a myriad of other pulmonary conditions, presenting with fever, nonproductive cough, pleuritic chest pain, and dyspnea [3]. This nonspecific symptomatology necessitates a high index of suspicion, particularly in individuals with known immunosuppression, to prompt timely diagnostic and therapeutic interventions.

Radiologically, PCP exhibits a wide array of features, from classic ground-glass opacities and pneumatoceles to less common findings such as cystic changes and rapid consolidation, contingent upon the patient's underlying immune status and prophylactic treatment history. Nodules formed from PCP can even progress to cavitary lesions with subsequent pneumothorax. The role of imaging, especially high-resolution computed tomography (CT), is pivotal in not only suggesting the diagnosis in the appropriate clinical context but also delineating the extent of pulmonary involvement and identifying potential complications [4].

By highlighting the varied clinical and radiologic presentations of PCP, this report aims to enhance the understanding and recognition of this potentially life-threatening infection in immunocompromised patients. The insights provided herein emphasize the importance of considering PCP in the differential diagnosis of pulmonary infections, thereby facilitating prompt and targeted therapeutic strategies to mitigate morbidity and mortality associated with this opportunistic pathogen.

## Case presentation

We present the case of a 25-year-old Latin male who presented to the emergency department complaining of a one-month history of chest pain, dyspnea, and a nonproductive cough, which recently escalated to include night sweats. The patient denied any symptoms of fever, chills, hemoptysis, or weight loss and reported no history of smoking or intravenous drug use. Having recently immigrated to the United States, he had no notable past medical or family history.

**Figure 1:**
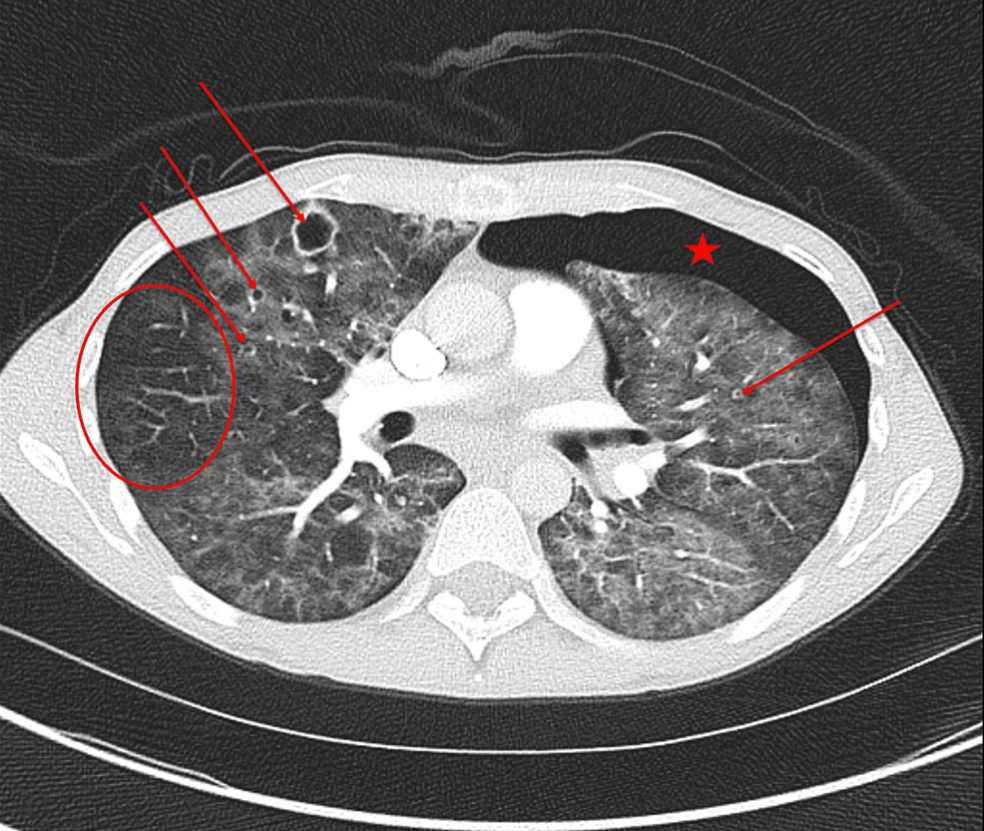
Chest CT axial section demonstrating bilateral diffuse ground-glass opacities, left pneumothorax (red star), subpleural sparing located in the right superior lobe (red circle), and multiple bilateral pneumatoceles located in the anterior segment of the right superior lobe and the anterior segment of the left superior lobe (red arrows).

Laboratory testing and diagnostic imaging, which included a complete blood count (CBC), basic metabolic panel (BMP), and chest radiograph, were obtained. Significant findings included leukocytosis and a moderate pneumothorax. He was subsequently managed with the insertion of a chest tube. Further evaluation through chest CT confirmed the pneumothorax (Figure 1) and revealed diffuse lung disease characterized by multiple thin- and thick-walled cystic lesions set against a backdrop of diffuse ground-glass opacities, with significant peripheral sparing evident.

Laboratory tests including toxicology screening and assays for COVID-19, streptococcus, and legionella antigens returned negative. The radiological findings were strongly suggestive of PCP. Additional testing confirmed that the patient was human immunodeficiency virus (HIV) positive, with a white blood cell count of 6.4 k/mcL and a critically low CD4 count of 1 cell/mm^3^, further reinforcing the suspicion of PCP.

A bronchoalveolar lavage was performed, and polymerase chain reaction (PCR) testing for *Pneumocystis jirovecii *returned positive. Based on the comprehensive clinical and radiologic evaluation, prompt intervention for PCP with targeted antibiotics, steroid therapy, and supportive care was initiated. This timely treatment allowed the patient to recover well over the next week.

## Discussion

PCP represents a life-threatening condition rarely encountered in immunocompetent individuals. It is predominantly observed in patients with AIDS but is also prevalent among those receiving immunosuppressive therapy for malignancies, those with rheumatologic disorders, and those receiving post-transplantation care [1,2]. Clinically, PCP manifests symptoms that overlap with various other pulmonary conditions, including fever, nonproductive cough, pleuritic chest pain, and dyspnea. Given this nonspecific symptomatology, a high index of suspicion is required, especially in patients with known immunosuppression, to facilitate prompt diagnostic and therapeutic measures [3].

The presentation of our patient was atypical, being previously healthy and immunocompetent with no history of malignancy, steroid use, or immunotherapy. Such clinical background often complicates the suspicion of PCP based solely on symptoms. In our case, the identification of specific radiological features consistent with PCP contributed to the diagnosis. High-resolution CT is superior to chest radiographs for this purpose and can help confirm or rule out PCP [5]. The hallmark of PCP on CT scans is a ground-glass opacity predominantly affecting the perihilar or mid zones (Figure 1), with peripheral sparing observed in approximately 40% of cases [4]. Moreover, thin-walled cysts, typically pneumatoceles, appear in up to 30% of cases and can precipitate pneumothorax following cyst rupture [6]. A retrospective analysis reported that spontaneous pneumothorax occurred in 86% of patients with PCP [7]. Intriguingly, pneumothorax was the initial presentation in our patient, likely stemming from unresolved PCP given his prolonged symptoms of approximately one month. Despite the relatively low prevalence of PCP in industrialized countries such as the United States, the radiological signs were correctly associated with PCP, enabling timely HIV and PCP testing and an accurate diagnosis [8]. Early recognition of PCP facilitated swift treatment, leading to the patient's recovery without serious complications.

The definitive diagnosis of PCP is confirmed through the detection of trophozoites by immunofluorescent stains or PCR. In cases where bronchoscopy is not feasible, serum levels of (1-3)-β-D-glucan (Wako assay), a component of fungal cell walls, have been shown to be a specific test aiding in the diagnosis of PCP [9]. Fortunately, our patient underwent a successful bronchoalveolar lavage, and the diagnosis of PCP was supported by a positive PCR result. Previous meta-analyses have noted significantly higher mortality rates associated with delays from symptom onset to diagnosis [10]. Therefore, rapid and non-invasive diagnostic tests such as the (1-3)-β-D-glucan assay could minimize diagnostic delays, potentially reducing mortality rates, and should be considered especially in patients who, despite appearing immunocompetent, present with symptoms or radiological indicators suggestive of PCP [11].

## Conclusions

This case highlights the critical importance of considering PCP in the differential diagnosis of pulmonary symptoms regardless of their apparent immune status. The occurrence of PCP in a seemingly healthy young individual without a known history of immunosuppression underscores the need for a comprehensive diagnostic approach, including detailed serologic and radiographic examinations when patients present with nonspecific respiratory symptoms. Our patient’s presentation with a pneumothorax, along with the discovery of HIV infection and critically low CD4 counts, exemplifies the potential complexity and severity of this infection even in industrialized settings where the incidence is low.

The successful management of this case was facilitated by a prompt and thorough evaluation that integrated clinical suspicion with advanced imaging and confirmatory laboratory tests. This approach not only ensured an accurate diagnosis but also enabled effective treatment before the development of more severe complications. This case serves as a reminder of the evolving epidemiology of PCP and the necessity for health care providers to maintain vigilance for opportunistic infections in all patients presenting with respiratory distress, irrespective of their immunocompetent appearance. Early diagnosis and intervention remain paramount in improving outcomes for patients afflicted with this potentially fatal disease.
